# Myomaker and Myomixer Characterization in Gilthead Sea Bream under Different Myogenesis Conditions

**DOI:** 10.3390/ijms232314639

**Published:** 2022-11-24

**Authors:** Miquel Perelló-Amorós, Aitor Otero-Tarrazón, Violeta Jorge-Pedraza, Isabel García-Pérez, Albert Sánchez-Moya, Jean-Charles Gabillard, Fatemeh Moshayedi, Isabel Navarro, Encarnación Capilla, Jaume Fernández-Borràs, Josefina Blasco, Josep Chillarón, Daniel García de la serrana, Joaquim Gutiérrez

**Affiliations:** 1 Departament de Biologia Cel·lular, Fisiologia i Immunologia, Facultat de Biologia, Universitat de Barcelona, 08028 Barcelona, Spain; 2 Laboratory of Fish Physiology and Genomics, UR1037, INRAE, 35000 Rennes, France

**Keywords:** myomaker, myomixer, myogenesis, muscle regeneration, muscle growth, fish

## Abstract

Skeletal muscle is formed by multinucleated myofibers originated by waves of hyperplasia and hypertrophy during myogenesis. Tissue damage triggers a regeneration process including new myogenesis and muscular remodeling. During myogenesis, the fusion of myoblasts is a key step that requires different genes’ expression, including the fusogens *myomaker* and *myomixer*. The present work aimed to characterize these proteins in gilthead sea bream and their possible role in in vitro myogenesis, at different fish ages and during muscle regeneration after induced tissue injury. Myomaker is a transmembrane protein highly conserved among vertebrates, whereas Myomixer is a micropeptide that is moderately conserved. *myomaker* expression is restricted to skeletal muscle, while the expression of *myomixer* is more ubiquitous. In primary myocytes culture, *myomaker* and *myomixer* expression peaked at day 6 and day 8, respectively. During regeneration, the expression of both fusogens and all the myogenic regulatory factors showed a peak after 16 days post-injury. Moreover, *myomaker* and *myomixer* were present at different ages, but in fingerlings there were significantly higher transcript levels than in juveniles or adult fish. Overall, Myomaker and Myomixer are valuable markers of muscle growth that together with other regulatory molecules can provide a deeper understanding of myogenesis regulation in fish.

## 1. Introduction

The skeletal muscle is a large and complex tissue formed by long multinucleated cells called myofibers that are the functional units of the locomotor system in vertebrates. The process by which the skeletal muscle is formed is known as myogenesis, where mesenchymal stem cells are committed to the muscle lineage as myoblasts, undergoing a process that involves proliferation and cell fusion events [1,2]. Such a complex cellular process is finely regulated by a series of highly conserved master transcription factors, known as myogenic regulatory factors (MRFs), which coordinate the expression of the required molecular machinery and structural components of the muscle. This myogenic program can occur during the embryonic development of the muscle, but also during adulthood in response to challenging conditions or tissue damage [3]. Hence, in animal production, to have a complete understanding of myogenesis may help to improve muscle growth and its remodeling and recovery after an injury. This knowledge can serve as a base for further research towards a better flesh quality and thus an improvement in the production of healthy protein sources for human consumption. In fish, in contrast to mammals, myogenesis extends beyond the adult stage due to an indeterminate and continuous growth throughout their lives, which is made possible by mechanisms of hyperplasia and hypertrophy [4].

The MRFs, as well as other proteins from the paired box (Pax) and SRY-related HMG-box (Sox) family, control the expression of structural proteins, such as myosin heavy chain (MyHC) or the proteins that permit myoblast fusion, like the recently discovered muscle-specific proteins Myomaker and Myomixer. In vertebrates, muscle differentiation is based on the sequential activation of the MRFs, Pax and Sox molecules: first, the Myogenic Factor 5 (Myf5), the Myogenic Differentiation 1 Protein (MyoD) and Sox8 specify the myoblast for differentiation; next, Myogenin, Myogenic Factor 4 (Mrf4) and Pax7 participate in the late differentiation process and trigger the expression of myotube specific genes to form the multinucleated myofibers [5].

The muscle specific Myomaker and Myomixer proteins that participate in the regulation of myogenesis play a central role in cellular fusion, development, and regeneration of mammalian muscle [6]. Myomaker, first known as Tmem8c, is a highly conserved transmembrane protein in vertebrates. In mammals, *myomaker* encodes for a 221 amino acids (aa) protein [7], while in fish it has more variation. For instance, in rainbow trout (*Oncorhynchus mykiss*), *myomaker* encodes for 434 aa and in zebrafish (*Danio rerio*) for 221 aa [8,9]. Although most of the reported experiments were done in mammals, in all vertebrates studied, including fish, *myomaker* expression is fundamental in both embryogenesis and regeneration of adult skeletal muscle [8,9,10,11,12,13,14]. The expression pattern of *myomaker* is similar to *myod* and *myogenin* because its regulation is in fact carried out through these MRFs [15].

Indeed, the transcription factors MyoD and Myogenin bind to the two E-boxes present in the promoter of the *myomaker* gene to induce its expression [12,15]. Several studies in mice have shown that *myomaker* expression was maximal during myoblast fusion and that the loss of *myomaker* inhibited cell fusion during myogenesis [11]. Thus, all these data confirmed the involvement of Myomaker in this process of myotubes formation. In fish, most of this research was done in salmonids or zebrafish. In rainbow trout, *myomaker* is expressed during embryogenesis and muscle regeneration after an injury, where the maximal expression is at the stage of the myoblast fusion [9], and in zebrafish, *myomaker* expression is restricted to embryogenesis [8]. Thus, further studies in non-salmonid fish are needed to understand the role of Myomaker in myogenesis and in adult teleost muscle regeneration.

Myomixer, also called Myomerger or Minion, is a weakly conserved transmembrane protein in vertebrates. In mammals, *myomixer* encodes for an 84 aa protein [16]. In fish, Myomixer is a 75 aa protein in zebrafish [16] and a 77 aa peptide in rainbow trout [14]. *myomixer* is expressed during embryogenesis, and muscle regeneration and myoblast differentiation in mice and trout [10,14,16]. Similarly to *myomaker*, MyoD and Myogenin regulate the transcription of *myomixer* by binding to the three E-boxes of its promoter during myogenesis [13]. A recent study observed that the lack of *myomixer* produced a defect in the process of cell fusion throughout myogenesis, confirming the involvement of Myomixer during myoblast fusion [10].

The expression pattern during embryogenesis and muscle regeneration of both *myomaker* and *myomixer* is very similar, which originated from an initial theory of a physical interaction between both proteins (Reviewed by Chen and coworkers [6]). Nevertheless, a recent bibliography demonstrated that knocking out either *myomaker* or *myomixer* completely impaired myoblast fusion, but not myoblast differentiation, thus the coexistence of Myomaker (in both cells that are going to fuse) and Myomixer (in at least one of the cells) is necessary and enough to promote cell fusion. However, Chen and coworkers [6] later refuted the theory of the direct physical interaction between Myomaker and Myomixer and proved that both proteins are essential, but have independent roles in myoblast fusion in the studied species [6].

Muscle regeneration, in vitro myogenesis or the comparison of different growth stages offer useful models to approximate to the functions of MRFs, Myomaker and Myomixer, and their relationships in muscle growth regulation. Thus, the objective of this study was first to characterize *myomaker* and *myomixer* genes in gilthead sea bream, and second, to investigate their expression during in vivo muscle regeneration after an induced injury, throughout in vitro myogenesis, and at different stages of fish muscle growth.

## 2. Results

### 2.1. Myomaker and Myomixer Characterization

**Figure 1:**
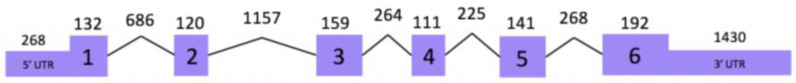
Structure of the *S. aurata myomaker* gene. The size of exons including the UTRs (purple boxes) and introns (lines) are indicated in the number of nucleotides.

A search in GenBank was performed to identify the gilthead sea bream *myomaker* mRNA (XM_030418477.1) while the genomic sequence of *myomaker* was found in the gilthead sea bream genome deposited in the Ensembl (ENSSAUG00010019449). Two ancient paralogues of *myomaker* were identified, named *pgap6* (ENSSAUG00010014619) and *tmem8b* (ENSSAUG00010020348), with low homology to *myomaker*, 33.19 and 30.60%, respectively. This search also revealed a single transcript (ENSSAUT00010049102.1) that apparently contained only 5 exons encoding a 232 aa protein. However, the alignment between the cDNA and the genomic sequence revealed that the automatic exon finding algorithm of the Ensembl included the exon 6 sequence inside the 3′ UTR. Thus, it was determined that the *myomaker* gene was situated in chromosome 5 and contained 6 exons encoding a protein of 285 aa (Figure 1). The gilthead sea bream Myomaker protein (XP_030418477.1) shared 89.50% identity with the zebrafish Myomaker (NP_001002088.1) protein; 81.75% identity with the rainbow trout Myomaker protein (XP_021476828.1) and 71.56% with the mouse Myomaker protein (NP_079652.1), pointing out a good conservation of the Myomaker sequence across vertebrates.

The gilthead sea bream *myomixer* sequence was provided by the CCMAR Sequence Server. The cDNA sequence of *myomixer* was blasted against the gilthead sea bream genome deposited in the Ensembl to find its genomic sequence (ENSSAUG00010011859, no paralogs identified). The *myomixer* gene is located in the chromosome 15 and has one single transcript (ENSSAUT00010028952.1) containing only 1 exon encoding a 75 aa protein. The gilthead sea bream Myomixer protein shares 70.67% identity with the zebrafish Myomixer protein (P0DP88.1); 69.33% with the rainbow trout Myomixer protein (QII57370.1), and 33.33% with the mouse Myomixer protein (NP_001170939.1). In this case, the conservation of Myomixer is low, either among fish species or with other vertebrates.

**Figure 2:**
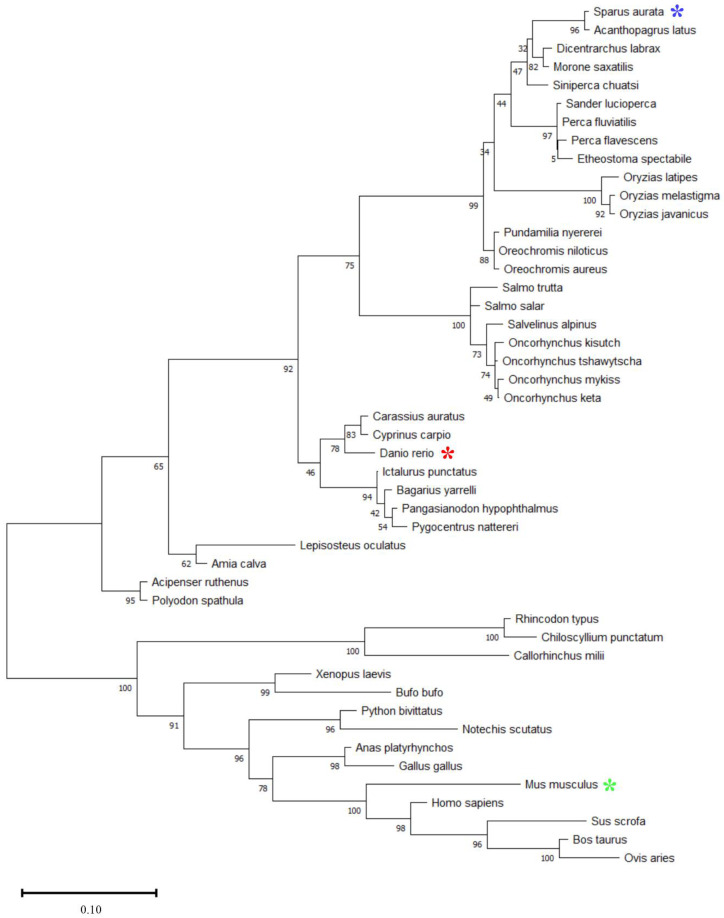
Phylogenetic analysis of Myomaker protein sequences among mammals, birds, reptiles, amphibians, and fish. Multiple alignment of whole protein sequences was done through the MAFFT tool (https://mafft.cbrc.jp/alignment/server/, accessed on 12 February 2020) with the iterative refinement L-INS-i method. The phylogenetic tree was developed with the Maximum Likelihood phylogeny and the JTT+G substitution model using the MEGA 11 v11.0.1.10 program. The numbers in the tree nodes represent the percentage of the bootstrap values after 500 replicates. The study species (*Sparus aurata*), as well as the model species *Danio rerio* and *Mus musculus* are marked with blue, red and green asterisks.

**Figure 3:**
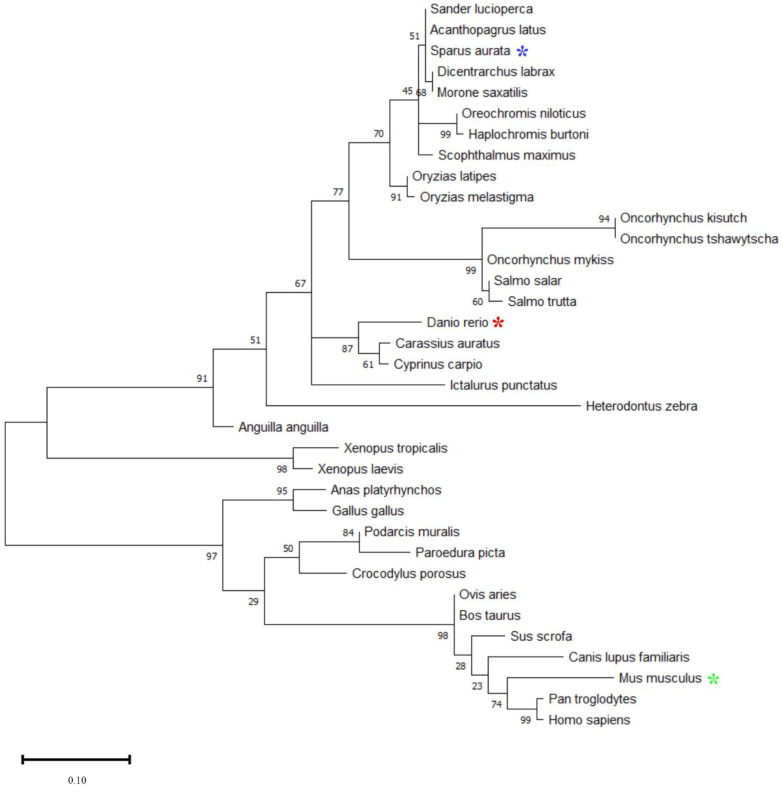
Phylogenetic analysis of Myomixer protein in mammals, birds, reptiles, amphibians, and fish. Multiple alignment of whole protein sequences was done through the MAFFT tool (https://mafft.cbrc.jp/alignment/server/, accessed on 12 February 2020) with the progressive G-INS-1 method. The phylogenetic tree was developed with the Maximum Likelihood phylogeny and the JTT+G substitution model using the MEGA 11 v11.0.1.10 program. The numbers in the tree nodes represent the percentage of the bootstrap values after 500 replications. The study species (*Sparus aurata*), as well as the model species *Danio rerio* and *Mus musculus* are marked with blue, red and green asterisks.

The phylogenetic analysis of the Myomaker and Myomixer aa sequences is shown in the Figure 2 and Figure 3. In both cases, a clear evolution of the proteins is observed across vertebrates, from fish to mammals. The Myomaker and Myomixer gilthead sea bream sequences are more closely related to other perciformes, such as other Sparidae species (*Acanthopagrus latus*) and other species such as the European sea bass (*Dicentrarchus labrax*). Myomixer diverged notably more than Myomaker. Moreover, the Myomaker protein sequences in fish have a great disparity in length (Figure 2). In gilthead sea bream, Myomaker is a 285 aa protein, while in salmoniforms it ranges from 400 aa in brown trout (*Salmo trutta*) and up to 477 in chinook salmon (*Oncorhynchus tshawytscha*). In chondrichthyans, the sequence of Myomaker is the smallest, having 218 aa similar to the Myomaker protein sequences in terrestrial vertebrates (amphibians, reptiles, birds and mammals), which vary between 220 and 221 aa.

In contrast, the length of the Myomixer protein sequences (Figure 3) diverged less than Myomarker among the different vertebrate species. In most fish, Myomixer has 75 aa, as in *S. aurata*. However, some salmonids, such as *O. kisutch*, have a sequence of 99 aa. In terrestrial vertebrates, the sequences range from 62 aa in birds to 108 aa in the reptile *Paroedura picta*. In mammals, Myomixer remains at 84 aa. 

### 2.2. Myomaker and Myomixer Tissue Screening

**Figure 4:**
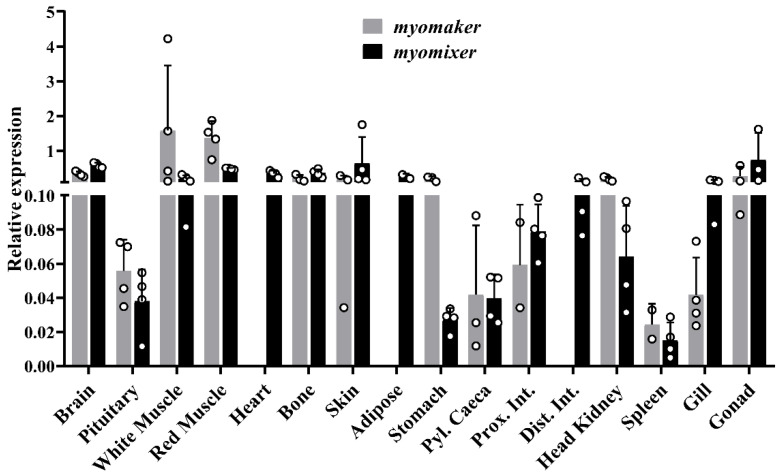
Bar plots of *myomaker* and *myomixer* relative gene expression in several tissues of 200 g gilthead sea bream. White-filled circles represent individual relative gene expression values. Data are presented as means + SEM (*n* = 4).

Figure 4 shows the *myomaker* and *myomixer* gene expression in gilthead sea bream tissues. The *myomaker* gene was mostly expressed in white and red muscle at similar levels while in the rest of the tissues, the mRNA levels were very low. In contrast, the *myomixer* gene showed expression in white and red muscle, as well as in skin, heart, brain, adipose tissue, bone, and gonad with relatively high levels. Both the *myomaker* and *myomixer* transcript levels in these tissues were also confirmed in the agarose gel after a qPCR. In the other tissues, the expression of *myomixer* was insignificant. Hence, both genes appear to be expressed, in addition to muscle, in a variety of extra muscular tissues at different intensities.

### 2.3. Regeneration Study

#### 2.3.1. Histological Analysis

**Figure 5:**
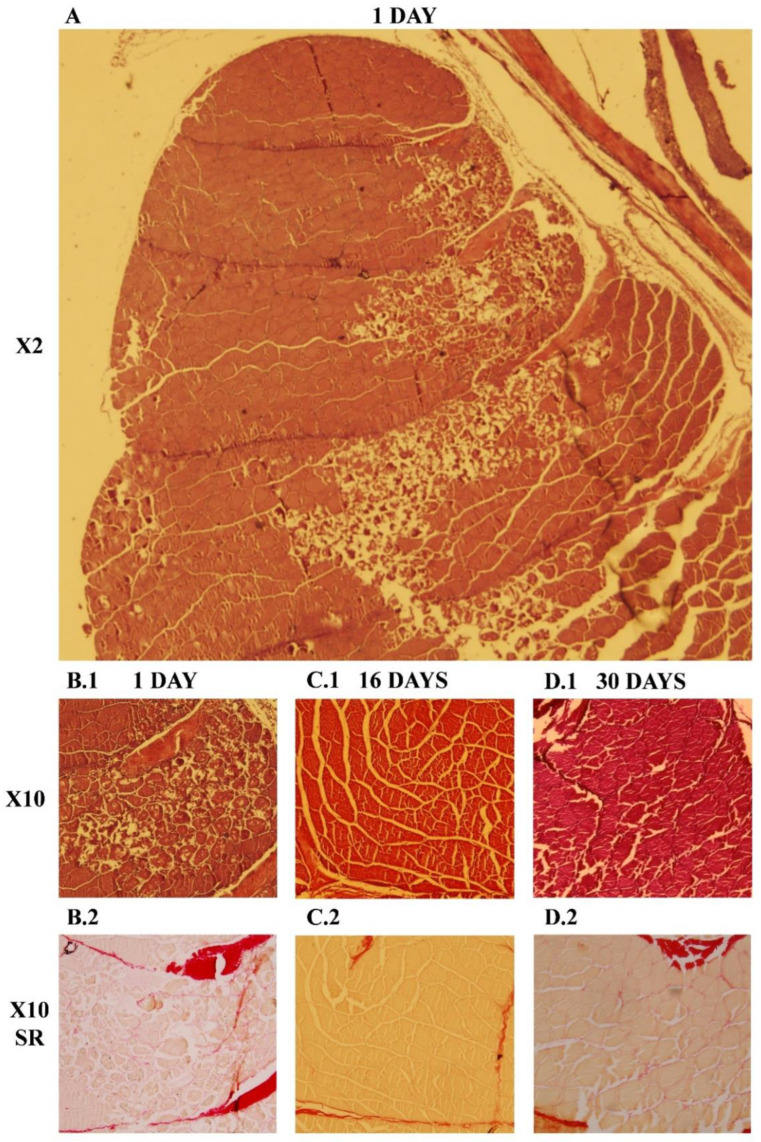
Histological evaluation of the muscle regeneration process at 1 (**B.1**,**B.2**); 16 (**C.1**,**C.2**) and 30 (**D.1**,**D.2**) days post injury. Muscle sections (7–10 µm) were stained with hematoxylin/eosin (**A**,**B.1**,**C.1**,**D.1**) and Sirius red (**B.2**,**C.2**,**D.2**). X2 and X10 means 2× and 10× objective magnification, respectively. Ocular magnification was 10×. SR = Sirius red staining.

The histological evaluation revealed that 24 h after the injury (Figure 5(A–B.2)), the tissue damage was clearly visible, with death of the damaged myofibers, important infiltration of immune cells and enlarged collagen deposition in the myoseptum close to the injury site as a sign of inflammation. At day 16, no signs of necrotic tissue were observed, while new small fibers appeared as the inflammatory phase might have ended. Finally, at 30 days post-injury (Figure 5(C.1,C.2)), no signs of the injury or fibrosis were observed, and the tissue presented a normal morphology.

#### 2.3.2. Myomaker and Myomixer

**Figure 6:**
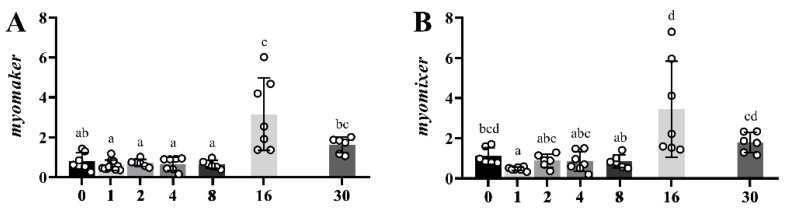
Bar plots of relative gene expression of *myomaker* (**A**) and *myomixer* (**B**) along the regeneration experiment in white skeletal muscle from day 0 (time of injury) to day 30. White-filled circles represent individual relative gene expression values. Data are presented as means ± SEM (*n* = 10). Different letters indicate significant differences (*p* < 0.05).

Figure 6 shows a comparative expression profile of *myomaker* (A) and *myomixer* (B) genes during the regeneration period in white muscle of gilthead sea bream from the time of injury (day 0) to 30 days later. Thus, after a stable period with almost constant values until day 8, a significant peak (three-fold) at day 16 was observed following a decrease up to day 30.

#### 2.3.3. MRFs

**Figure 7:**
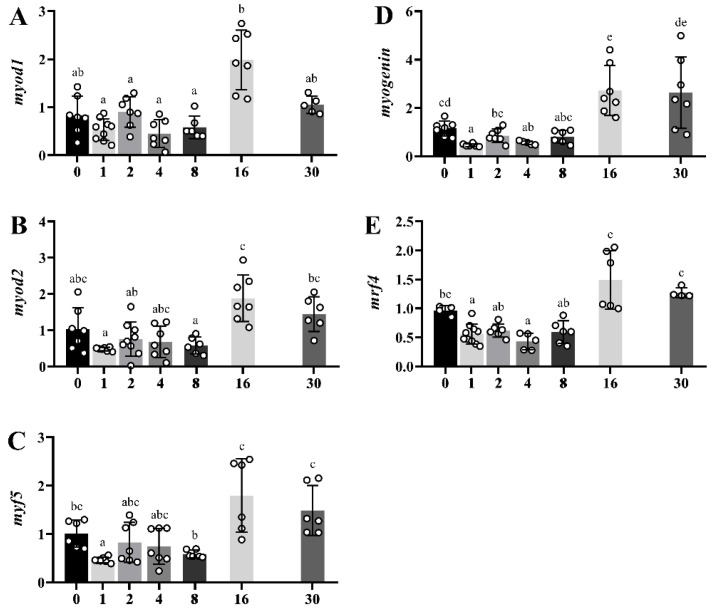
Bar plots of relative gene expression of *myod1* (**A**), *myod2* (**B**), *myf5* (**C**), *myogenin* (**D**) and *mrf4* (**E**) along the regeneration experiment in white skeletal muscle from day 0 (time of injury) to day 30. White-filled circles represent individual relative gene expression values. Data are presented as means ± SEM (*n* = 10). Different letters indicate significant differences (*p* < 0.05).

The different myogenic genes (*myod1*, *myod2*, *myf5*, *myogenin* and *mrf4*,) presented a similar profile during muscle regeneration with a maximum peak of expression 16 days after injury, maintaining high levels up to day 30 (Figure 7A–F). Hence, in this regeneration model in gilthead sea bream, the myogenic activity was at a maximum around 16 days post-injury with an overall downregulation trend at day 30.

### 2.4. In Vitro Myogenesis

#### 2.4.1. Myomaker and Myomixer

**Figure 8:**
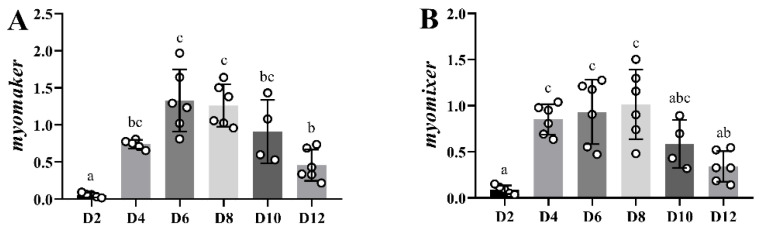
Bar plots of relative gene expression of *myomaker* (**A**) and *myomixer* (**B**) along the primary culture of myoblasts. White-filled circles represent individual relative gene expression values. Data are presented as means ± SEM (*n* = 6). Different letters indicate significant differences (*p* < 0.05).

The expression of *myomaker* and *myomixer* increased significantly already at day 4 of culture during in vitro myogenesis of gilthead sea bream, reaching its maximum level at day 6, followed by a progressive decrease, which became significant at day 12 (Figure 8).

#### 2.4.2. MRFs

**Figure 9:**
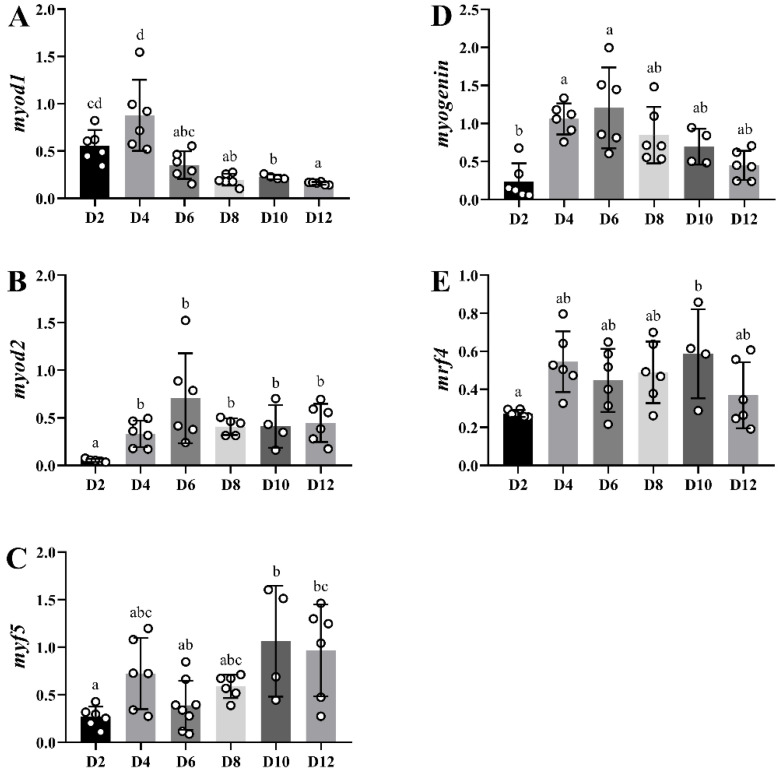
Bar plots of relative gene expression of *myod1* (**A**), *myod2* (**B**), *myf5* (**C**), *myogenin* (**D**) and *mrf4* (E) along the primary culture of myoblasts.White-filled circles represent individual relative gene expression values. Data are presented as means ± SEM (*n* = 6). Different letters indicate significant differences (*p* < 0.05).

*myod1* and *myod2* gene expression levels presented their earliest peak at day 4 and day 6, respectively. Then, in the case of *myod1*, levels progressively diminished, the decrease being significant at day 6 and reaching the lowest values of expression at day 12. *myod2* expression decreased after its peak, although not significantly, maintaining a plateau until day 12 (Figure 9A,B). Moreover, *myf5* had a slight peak of expression at day 2 (*p* = 0.104), to then show significantly higher values at days 10 and 12 in comparison to day 2 expression levels (Figure 9C). *myogenin* expression increased rapidly showing significant changes at day 4, followed by a progressive decrease from day 6 until day 12 (Figure 9D). The *mrf4* expression showed a tendency to increase already at day 4 (*p* = 0.062), reaching a significant upregulation only at day 10 (Figure 9E). Thus, in the proposed culture conditions and at this specific fish age, the proliferative phase of myogenesis spans from days 1 to 6, overlapping with the myoblast fusion stage, with the first myotubes appearing at day 4 of culture. Hence, from day 4 onwards, the proliferation and differentiation processes occur simultaneously, even at the more advanced stages of culture development.

### 2.5. Myomaker and Myomixer at Different Growth Stages

**Figure 10:**
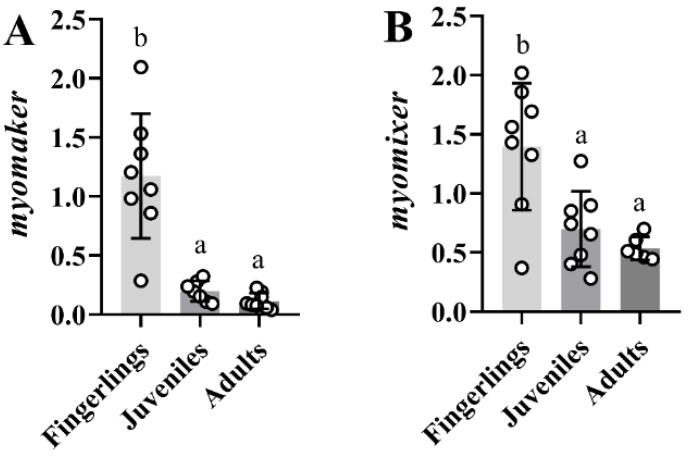
Bar plots of relative gene expression of *myomaker* (**A**) and *myomixer* (**B**) in white skeletal muscle of three different sizes of gilthead sea bream. White-filled circles represent individual relative gene expression values. Data are presented as means ± SEM (*n* = 6). Different letters indicate significant differences (*p* < 0.05).

Figure 10 shows the expression of *myomaker* (A) and *myomixer* (B) in the white muscle of fingerlings, juveniles, and adults of gilthead sea bream. Both genes showed a progressive decrease with the age of the fish. Thus, fingerlings presented the maximum gene expression levels, in juveniles those decreased significantly, and adults showed very low levels of *myomaker* and *myomixer,* although differences in gene expression were only significant in comparison to fingerlings.

## 3. Discussion

The muscle specific Myomaker protein that controls myoblast fusion was initially found in mice as a 221 aa protein, and it was described to have a similar transcription profile as those of *myod* and *myogenin* [11]. In fish, Myomaker was first described in zebrafish [8], and recently it has been characterized in rainbow trout [9] and yellowfin seabream (*Acanthopagrus latus*) [17]. In all three species, the gene is structured in 6 exons, but differences in the length of the protein are marked, being as long as 434 aa in rainbow trout [9], while just 285 aa in non-salmonid species such as yellowfin seabream [17] and gilthead sea bream. The protein sequence alignment between mice, rainbow trout and gilthead sea bream indicated that the N-terminal half of the rainbow trout Myomaker was similar to the mice and gilthead seabream sequences, while the C-terminal half did not have homology with any known motifs [9]. The phylogenetic analysis showed that Myomaker is a well conserved protein across vertebrate organisms, from fish to mammals [9,11]. The gilthead sea bream Myomaker protein presented a homology of 89%, 71% and 81% with zebrafish, mouse and the N terminal domain in rainbow trout, respectively. Moreover, a clear evolution among fish species was observed and the gilthead sea bream Myomaker sequence proved to be more closely related to other perciform species, such as the European sea bass (*D. labrax*), the beloniform species, such as *Oreochromis latipes*, or the salmonids.

The *myomixer* gene was also firstly identified in mice, contained a single exon and encoded an 84 aa protein. Additionally, the mice *myomixer* gene had another transcript form, less conserved, which had 3 exons and yielded a protein of 108 aa [10,13], but until today, a single *myomixer* transcript has been described in the few fish species where this gene has been studied. Indeed, the only described gilthead sea bream *myomixer* transcript (ENSSAUT00010028952.1) that was found in the gilthead sea bream genome deposited in Ensembl contained only one exon that encoded a 75 aa protein, similarly to the rainbow trout *myomixer* (77 aa), although in this salmonid species, the gene is structured in two exons [14].

The Myomixer protein sequence showed weak cross-species conservation, with mammals and fish sharing only 36% identity [14,16]. Among fish, the Myomixer gilthead sea bream showed a homology of 70.67% and 69.33% with the zebrafish and rainbow trout Myomixer protein sequences, respectively [14]. Nevertheless, the crucial AxLyCxL motif of the Myomixer micropeptide presented a high conservation across vertebrates. As in the case of Myomaker, the gilthead sea bream Myomixer sequence was more related to that from other perciformes, such as the European sea bass, beloniformes or salmoniformes. Other phylogenetic analysis performed in gilthead sea bream, such as those for the Myogenin and Preproghrelin proteins, also showed that these molecules evolved in the same way, being closer to other perciforms, while being more distant to salmoniformes or cypriniformes [18].

Regarding the tissue screening, on one side, the gene expression of *myomaker* in gilthead sea bream showed a narrow distribution among tissues, being expressed mainly in white and red skeletal muscles. Such expression distribution was also observed in rainbow trout [9], while in yellowfin seabream it presented a wider expression across the tissues [17]. On the other hand, the *myomixer* pattern of expression appeared to be more divergent among species, while in the rainbow trout and mice its expression was mostly restricted to white and red skeletal muscles [13,14]; in yellowfin seabream [19] and adult gilthead sea bream its expression was also detected in several other tissues ¡. It is noteworthy, however, that both *myomaker* and *myomixer* genes in humans have a broad expression through a large list of cell types and tissues according to the GeneCards database (GC09M133515 and GC06P087997, accessed on 26 September 2022). Hence, to date there is no specific literature that focuses on understanding whether these two genes play a significant physiological role in the rest of the tissues, such as regulating membrane fusion or other membrane processes within the cells.

While in mammals the regenerative myogenesis is a well-known process, in fish, some aspects remain unclear. Muscular regeneration in gilthead sea bream after a provoked injury was first studied by Rowlerson and coworkers [20], where the histological analysis showed a high cellular proliferation with a greater deposition of dense connective tissue and new small myofiber formation around the lesion site by 7–11 days after the injury was made. After 21 days, groups of small fibers were evident at the injury site, highlighting an incomplete regenerative process at this time. In the present study, we proved that in gilthead sea bream, at day 1, the injury was easily observed by the naked eye, and the histological evaluation revealed a massive death of the muscle fibers longitudinally to the injury point with a strong cell infiltration. After 30 days of regeneration, the morphology of the white muscle fibers at the optical microscopy level was highly similar to the undamaged muscle, with only slight signs of recessing inflammation [20]. However, the fiber organization of the rainbow trout muscle after 20 and 30 days of a mechanical injury remained altered, with a high deposit of connective tissue embedded with small muscle fibers. Such results point out that at this time, the regeneration is far from being completed in salmonids [9,21] while in gilthead sea bream appears to be almost completed.

At a transcriptional level, there were appreciable differences between rainbow trout and gilthead sea bream, indicating a faster regenerative process in the latter. In mice, both *myomaker* and *myomixer* expression was strongly detected in regenerating muscle 3 days after the injury, and then it rapidly decreased in less than 2 days when the new myofibers were formed, which indicated that both proteins are essential for muscle regeneration [10,11]. The regulation of the expression of both genes is mediated by two E-boxes in the promoter, which are described as targets of MyoD and Myogenin [15]. Furthermore, specifically knocking out *myomaker* in the mice satellite cells in vivo completely impaired myoblast fusion, thus resulting in a complete blocking of muscle regeneration [12]. In fish, the implication of Myomaker and Myomixer in muscle regeneration has only been studied recently in rainbow trout, where the expression of both genes drastically increased only at 30 days post injury, along with *myogenin* [9,14], confirming as well the incomplete nature of the regenerative process after 30 days in salmonids. In the gilthead sea bream, the onset of the expression of *myomaker*, *myomixer* and all the MRFs started between 8- and 16-days post injury and were strongly upregulated at day 16, while most of the genes were decreasing at day 30, thus confirming that gilthead sea bream regenerates faster than trout.

The differences observed between the two species regarding the moment when the new myofibers were formed during myogenesis could be due to the distinct metabolic rates being higher in the 15 g gilthead sea bream juveniles reared at 21–23 °C compared to the 1 kg rainbow trout adults reared at 10–15 °C [9,14,22]. Overall, these data support the importance of both Myomaker and Myomixer in the regenerative process of skeletal muscle in gilthead sea bream.

The in vitro myogenesis in gilthead sea bream has been deeply characterized by our group [5,23,24,25,26,27,28]. In the present study, both *myomaker* and *myomixer* presented a highly parallel expression pattern, with an important upregulation at days 4 and 6 to then decrease progressively from day 8 to day 12. The current results coincide with those obtained in rainbow trout in vitro myoblasts, where the expression of both *myomaker* and *myomixer* increased progressively between days 4 and 6 of culture [9,14].

The MRFs expression during in vitro gilthead sea bream myogenesis was well described by our group [5,23,24,25], and the results of the present study are consistent with the literature. The *myod1* and *myf5* early peaks of expression agree with the function of these transcription factors at the onset of myogenesis in conjunction with *myod2*, which classically appears more delayed than *myod1* [29,30]. The upregulation of *myogenin* between days 4 and 6 followed by a progressive decrease was also observed by García de la serrana and coworkers [24] coinciding with its role in promoting myoblast differentiation and progress. Then, a peak of *mrf4* was observed at day 10, supporting this being a factor more involved in the finalization and maturation of myotubes. Thus, the high parallelism between *myogenin*, *myomaker* and *myomixer* expression confirms the role of these two novel fusogens in the later stages of myogenesis in gilthead sea bream and, in fact, their interaction with MyoD and Myogenin could be explained by the presence of E-boxes in their promoters, as pointed out in fish and mammalian models [9,15,19].

Finally, the comparison of *myomaker* and *myomixer* transcript levels in fish at different ages suggests that both factors play a more active function at the stage of fingerlings. These results are in agreement with the findings described in rainbow trout [9,14], where the expression of *myomixer* and *myomaker* were maximum at the stage of embryo, decreasing progressively at 15, 150 and 1500 g. All this information supports the role of both factors in somitogenesis or strong growing stages such as in fingerlings, to then decrease in juveniles or adults where the level of hyperplasia is less important. Thus, in mouse and zebrafish, the expression of *myomixer* declines soon after somitogenesis [10,16], whereas in trout its expression is maintained throughout post-larval growth, i.e., in fry, juvenile and to a lesser extent in mature fish.

Overall, the present results support that Myomaker and Myomixer play an important role in gilthead seabream not only during developmental myogenesis, especially at the second part of the process, when the myocytes differentiation takes place, but also during regenerative myogenesis, where their upregulation takes place only after 16 days of recovery, pointing out their role during the later differentiation stages. Therefore, our results contribute understanding the role of Myomaker and Myomixer in a fish species of undetermined growth, normally living in high temperature waters and with high interest for aquaculture.

## 4. Materials and Methods

### 4.1. Fish Maintenance and Distribution

In order to perform the muscle regeneration experiment, 140 gilthead sea bream (*Sparus aurata*) juveniles (initial body weight: 15.4 ± 3.5 g; initial length: 8.7 ± 0.6 cm) were obtained from a commercial hatchery (Piscimar, Borriana, Spain) and were placed in and adapted to the fish facilities of the Faculty of Biology (University of Barcelona). Fish were randomly distributed in three 200 L seawater tanks (46–47 fish/tank). Four gilthead sea bream adults of 214 ± 12.13 g were reared in one 200 L tank for a tissue screening analysis of Myomaker and Myomixer and, additionally, three groups of eight gilthead sea breams weighing 5.88 ± 0.51 g; 122.38 ± 2.31 g and 387.13 ± 41.9 g were reared in three 200 L tanks for an ontogenetic study of the expression of both genes. Each tank had a constant flux of 700 L/h in a seawater semi-closed recirculation system with a weekly water renewal of 20–30%, a salinity of 35–37‰, a constant temperature of 23 ± 1 °C and a photoperiod of 12 h light/12 h dark. Fish were fed *ad libitum* three times per day (9:00 a.m., 2:00 p.m., and 7:00 p.m.) with a commercial diet (Perla, Skretting, Burgos, Spain) and were kept in the described conditions for the acclimation period during the 2 weeks before the experiments. The study was carried out following the EU recommendations and the procedures established by the Spanish and Catalan governments. The protocol was approved by the Ethics and Animal Care Committee of the University of Barcelona (CEEA 37/20).

### 4.2. Myomaker and Myomixer Characterization

The *myomaker* mRNA (cDNA) sequence of *S. aurata* (XM_030418477.1) was obtained from the GenBank (release 235) (https://www.ncbi.nlm.nih.gov/genbank/, accessed on 10 February 2020) and the *myomixer* sequence was found in the CCMAR Sequence Server database [31]. The *myomaker* and *myomixer* cDNA sequences were blasted against the gilthead sea bream genome deposited in Ensembl (release 99) (https://www.ensembl.org/Sparus_aurata/Info/Index, accessed on 10 February 2020) to obtain the genomic sequence of both genes for its characterization. Primers for the amplification by real-time quantitative PCR (qPCR) of *myomaker* and *myomixer* cDNA (Table 1) were designed using the sequences mentioned above with the Primer3Plus software (http://www.bioinformatics.nl/cgi-bin/primer3plus/primer3plus.cgi, accessed on 10 March 2020). The forward primer of *myomaker* was designed in the exon 1–exon 2 junction to avoid the amplification of genomic DNA and the reverse primer was placed in exon 2. The primers of *myomixer* were designed on its single exon, so DNase I pretreatment of the RNA was necessary before reverse transcription. The quality of the primers was tested by using the NetPrimer software (http://www.premierbiosoft.com/netprimer/, accessed on 10 March 2020). The collection of *myomaker* and *myomixer* sequences from different species was performed through the BLAST databases (v.2.10.0) (https://blast.ncbi.nlm.nih.gov/Blast.cgi, accessed on 12 February 2020). The Unipro UGENE v33.0 software was used to obtain the predicted protein sequences from the nucleotide sequences. Multiple Myomaker and Myomixer sequence alignments were performed with the MAFFT tool (v.7) (https://mafft.cbrc.jp/alignment/server/, accessed on 12 February 2020). The iterative refinement method L-INS-i was used for the Myomaker sequence and the progressive method G-INS-1 was used for the Myomixer sequence. In both cases, a gap opening penalty of 1.53 (default settings) was used. The alignments were confirmed with the Unipro UGENE software (v.33.0). The phylogeny was developed with the Maximum Likelihood phylogenetic inference method of MEGA 11 (v.11.0.1.10). The JTT+G protein substitution model was used with a bootstrap value of 500.

### 4.3. Tissue Screening

Four 214 ± 12.13 g gilthead sea breams were deprived of food overnight, anesthetized with MS222 (100 mg/L) and weighed. Blood was then drawn from the caudal vein with a 1 mL sterile syringe and a 0.4- or 0.6-mm needle previously precoated with EDTA-Li to prevent blood clotting. For tissue collection, fish were slaughtered by cervical section of the spine and the following tissues were extracted: white muscle, red muscle, skin, heart, brain, adipose tissue, liver, spleen, hypophysis, kidney, gill, intestine, bone, pyloric caeca, stomach, and gonad. The tissues were introduced in RNase-free microtubes that were stored in liquid nitrogen during sampling and at the end at −80 °C until further analysis.

### 4.4. In Vitro Myogenesis: Primary Myoblast Culture

Since our first publication [32], our group has used the in vitro model of muscle cells to study the endocrine regulation of muscle function and the myogenesis process in fish. In the paper by Montserrat and coworkers [23], the detailed characterization of the in vitro development of gilthead sea bream myocytes was shown. Later, Garcia de la serrana and coworkers [24] analyzed the expression of the MRFs and other regulatory factors throughout in vitro myogenesis in the same species. Based on this experience, we used the in vitro model to study the expression of *myomaker* and *myomixer* along with the MRFs during the myogenic process; thus, both genes were analyzed during the course of the primary myoblast culture from the proliferative stage (days 0 to 4) and through the complete differentiation process (day 4 to 12).

The primary satellite cells from gilthead sea bream skeletal white muscle were isolated and cultured as myoblasts, as previously described [23,24,33]. A total of six independent cell isolations were performed as biological replicates and samples for gene expression were taken every two days after the satellite cells’ seeding.

### 4.5. Muscle Regeneration Experiment

The muscle regeneration experiment aimed to better understand the role of Myomaker and Myomixer in myogenesis after muscle injury. To do that, 140 gilthead sea breams were divided into two groups: injured fish (I) and control fish (C). First, gilthead sea bream juveniles were all anesthetized with MS222 (100 mg/L) and then measured and weighed. To identify the fish, a passive integrated transponder (PIT) tag (ID-100A (1.25) Nano transponder; Trovan Electronic Identification Systems, Madrid, Spain) was inserted subcutaneously into the left anterior epaxial muscle just below the first radius.

Subsequently, an injury was performed with a 2.108 mm (14 G) diameter needle inserted vertically into the left epaxial muscle below the sixth radius to a depth of 1 cm. To know exactly where the needle was introduced, the tip of the sixth radius was cut. The wound was then healed with iodine alcoholic solution and the fish was allowed to recover in a separated small tank before being returned to its original tank. For more detailed information on the experimental procedure, on how the injury and the skeletal muscle sampling was made, see the Supplementary Material.

Samplings were done at days 0, 1, 2, 4, 8, 16 and 30 after the injury, in which white muscle was extracted. At each time point, fish were deprived of food overnight and 20 fish were randomly selected for sampling (4–5 injured fish/tank and 2 control fish/tank). Fish were first anesthetized, identified by reading the pig tag, weighed to note the changes on body weight, and then blood was drawn. For tissue extraction, all fish were slaughtered as mentioned previously. In injured fish, a section of the muscle was removed from the left side (injured), while the right side was also taken as a self-control for each fish. The size of the muscle extracted was 0.5 cm wide and 1 cm long just below the cut radius. All tissue samples were placed in RNase-free microtubes, which were stored in liquid N_2_ during sampling and then at −80 °C until further analysis. An additional group of two fish per tank were equally injured for histological analysis of the regenerative process at days 1, 16 and 30 post injury. For this purpose, blocks of 0.5 cm wide and 1 cm long were properly removed just below the cut radius and were fixed in 10% neutral-buffered formalin. Samples were dehydrated and embedded in paraffin. 7–10 µm tissue sections were obtained along the full muscle block with a rotary microtome (pfm, ROTARY 3003, Köln, Germany). For every pair of consecutive slides, one was stained with hematoxylin/eosin and the other was stained with Sirius red. All preparations were observed under a light microscope and photographed at different magnifications (Olympus PM10SP Automatic Photomicrography System). All reagents for histology staining were purchased from Merck (Mollet del Vallès, Spain).

### 4.6. RNA Extraction, cDNA Synthesis and qPCR Analyses

In summary, to perform the gene expression analyses: 4 fish were sampled for the tissue screening; 10 fish were used for the muscle regeneration study per sampling point; 6 individual wells per time point were sampled for the in vitro model and 8 fish for the ontogenesis experiment per age stage were sampled. For RNA extraction, 1 mL of TRI Reagent Solution^®^ (Applied Biosystems, Alcobendas, Spain) was added to the samples (around 0.04 g for liver and 0.1 g for the rest of the tissues, whenever possible). Samples were homogenized with Precellys Evolution^®^ (Bertin Instruments, Montigny-le-Brettoneux, France) adjusting the protocol depending on the hardness and elasticity of the tissue. Here below, RNA extraction was performed following the manufacturer’s instructions for the TRI Reagent Solution^®^. The final concentration of each sample was obtained using a Nanodrop 2000TM (Thermo Scientific, Alcobendas, Spain). RNA integrity was confirmed in a 1% agarose gel (*m*/*v*) stained with SYBR-Safe DNA Gel Stain^®^ (Life Technologies, Alcobendas, Spain). For cDNA synthesis, 1 μg of total RNA was treated with DNase I Amplification Grade^®^ (Life Technologies, Alcobendas, Spain) to remove all genomic DNA. Reverse transcription was carried out with the First Strand cDNA synthesis Transcriptor Kit^®^ (Roche, Sant Cugat del Valles, Spain) following the manufacturer’s recommendations. According to the requirements of the MIQE guidelines [34], the mRNA transcript levels of the genes were analyzed by qPCR using the CFX384TM Real-Time System (Bio-Rad, El Prat de Llobregat, Spain). The analysis was performed in a final volume of 5 μL, containing 2.5 μL of iTaq SYBR Green Supermix^®^ (Bio-Rad, El Prat de Llobregat, Spain), 0.125 μL of forward (250 nM) and reverse (250 nM) primers, 1 μL of cDNA from each sample and 1.25 μL of DEPC water. The reaction was performed in triplicate in 384-well plates (Bio-Rad, El Prat de Llobregat, Spain) under the conditions described by Salmerón and coworkers [35]. The qPCR consisted of (1) an activation phase of 3 min at 95 °C; (2) 40 cycles of 10 s at 95 °C, and 30 s at 55–68 °C (dependent of the melting temperature of the primers, Table 1); and (3) a melting curve from 55 °C to 95 °C that increased by 0.5 °C every 30 s. Before this analysis, the adequate cDNA dilution for each gene was determined by a dilution curve with a pool of samples. With this analysis, the specificity of the amplification, the absence of primers-dimers and the efficiency of the primers were also tested.

The expression level of each gene was calculated with the Pfaffl method [36] and was analyzed relative to the geometric mean of the reference genes (*rps18*, *rpl27* and *ef1a*). The reference genes, the most stable under different conditions, were confirmed with the GeNorm algorithm.

### 4.7. Statistical Analyses

Data were analyzed using IBM SPSS Statistics v.25 and were presented as mean ± standard error of the mean (SEM). Normal distribution was analyzed using the Shapiro-Wilk test and homogeneity of the variances (homoscedasticity) was assessed with a Levene’s test. If normal distribution and/or homoscedasticity was not found, data were transformed logarithmically. Differences were tested by Student’s *t*-test or one-way analysis of variance (ANOVA) and the post hoc Tukey’s HSD. If necessary, the nonparametric Kruskal Wallis test and the Games-Howell post, hoc were used. Different letters on the bars represent significant differences according to the post hoc tests. Additionally, a one-way ANOVA was performed to verify that the tank did not influence the measured parameters. Statistical differences were considered significant when *p* < 0.05.

## Figures and Tables

**Table 1:** Primers used in the Real-Time quantitative PCR analyses.

Gene	Primer Sequences (5′-3′)	Ta (°C)	Accession Number
*myf5*	F: CCATCCAGTACATCGAGAGCC R: ATCGCCCAAAGTGTCGTTCT	57	KJ524459
*myod1*	F: TTTGAGGACCTGGACCC R: CTTCTGCGTGGTGATGGA	60	AF478568.1
*myod2*	F: CACTACAGCGGGGATTCAGAC R: CGTTTGCTTCTCCTGGACTC	60	AF478569
*mrf4*	F: CATCCCACAGCTTTAAAGGCA R: GAGGACGCCGAAGATTCACT	60	JN034421
*myogenin*	F: CAGAGGCTGCCCAAGGTCGAG R: CAGGTGCTGCCCGAACTGGGCTCG	68	EF462191
*myomaker*	F: TTCACTGCGGTTTACCACGC R: CCCACATAGAGAGAGCTGTGCTG	60	XM_030418477.1
*myomixer*	F: TGCTGCGGTCCCTGGTTATC R: ACTCCTGGGATCGAATGCGG	60	LR537135.1
*ef1a*	F: CTTCAACGCTCAGGTCATCAT R: GCACAGCGAAACGACCAAGGGGA	60	AF184170
*rps18*	F: TGACGGAAGGGCACCACCAG R: AATCGCTCCACCAACTAAGAACGG	60	AY550956
*rpl27a*	F: AAGAGGAACACAACTCACTGCCCCAC R: GCTTGCCTTTGCCCAGAACTTTGTAG	60	AY188520

F: forward; R: reverse; Ta: annealing temperature; myf5: myogenic factor 5; myod1: myoblast determination protein 1; myod2: myoblast determination protein 2; mrf4: myogenic regulatory factor 4; ef1a: elongation factor 1 alpha; rps18: ribosomal protein s18; rpl27a: ribosomal protein l27a.

## Data Availability

All the data generated or analyzed during this study are included in this published article.

## Supplementary Materials

The supporting information can be downloaded at: https://www.mdpi.com/article/10.3390/ijms232314639/s1.
