# Supplementary material for: Myomaker and Myomixer Characterization in Gilthead Sea Bream under Different Myogenesis Conditions
Source: Int J Mol Sci. 2022 Nov 24;23(23):14639. doi: 10.3390/ijms232314639 (PMC9737248; doi:10.3390/ijms232314639)

International Journal of
Molecular Sciences

MDPI

Article

Myomaker and Myomixer Characterization in Gilthead Sea Bream under Different Myogenesis Conditions

Miquel Perelló-Amorós [1], Aitor Otero-Tarrazón [1], Violeta Jorge-Pedraza [1], Isabel García-Pérez [1], Albert Sánchez-Moya [1], Jean-Charles Gabillard [2], Fatemeh Moshayedi [1], Isabel Navarro [1], Encarnación Capilla [1], Jaume Fernández-Borràs [1], Josefina Blasco [1], Josep Chillarón [1], Daniel García de la serrana [1] and Joaquim Gutiérrez [1,*]

[1] Departament de Biologia Cel_lular, Fisiologia i Immunologia, Facultat de Biologia, Universitat de Barcelona, 08028 Barcelona, Spain
[2] Laboratory of Fish Physiology and Genomics, UR1037, INRAE, 35000 Rennes, France
* Correspondence: jgutierrez@ub.edu; Tel.: +34-934-021-532

SUPPLEMENTARY MATERIAL

The following supplementary images intend to provide more information on how the muscle regeneration model was carried out, especially the process of provoking the muscle injury as described in the materials and methods section of the manuscript. From up to down, and left to right, the pictures represent: size of the needle and stopping method for accurate length of the injury; internal muscle hemorrhage and injury visual evaluation at 2, 8 and 16 days post-injury; location and shape of the epaxial skeletal muscle block extracted from both, the non-injured (right) and injured (left) loins.

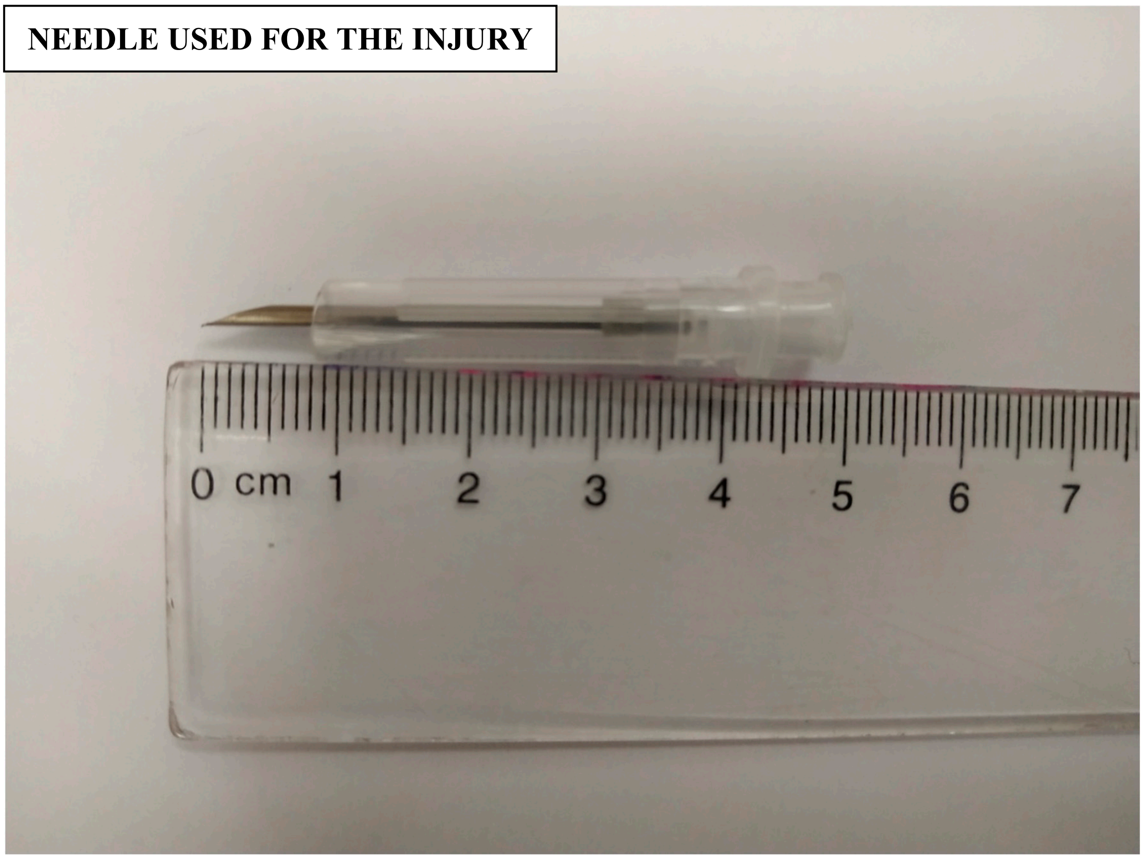
NEEDLE USED FOR THE INJURY
0 cm 1 2 3 4 5 6 7

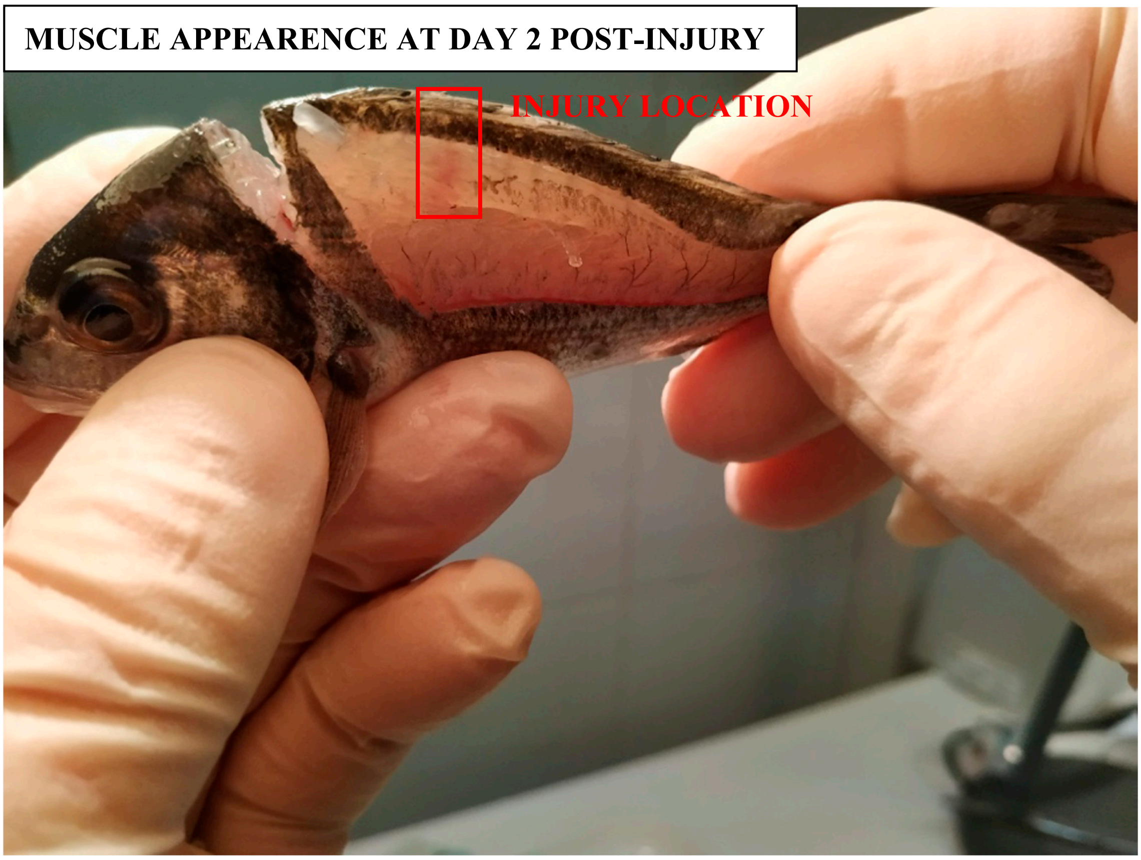
MUSCLE APPEARENCE AT DAY 2 POST-INJURY
INJURY LOCATION

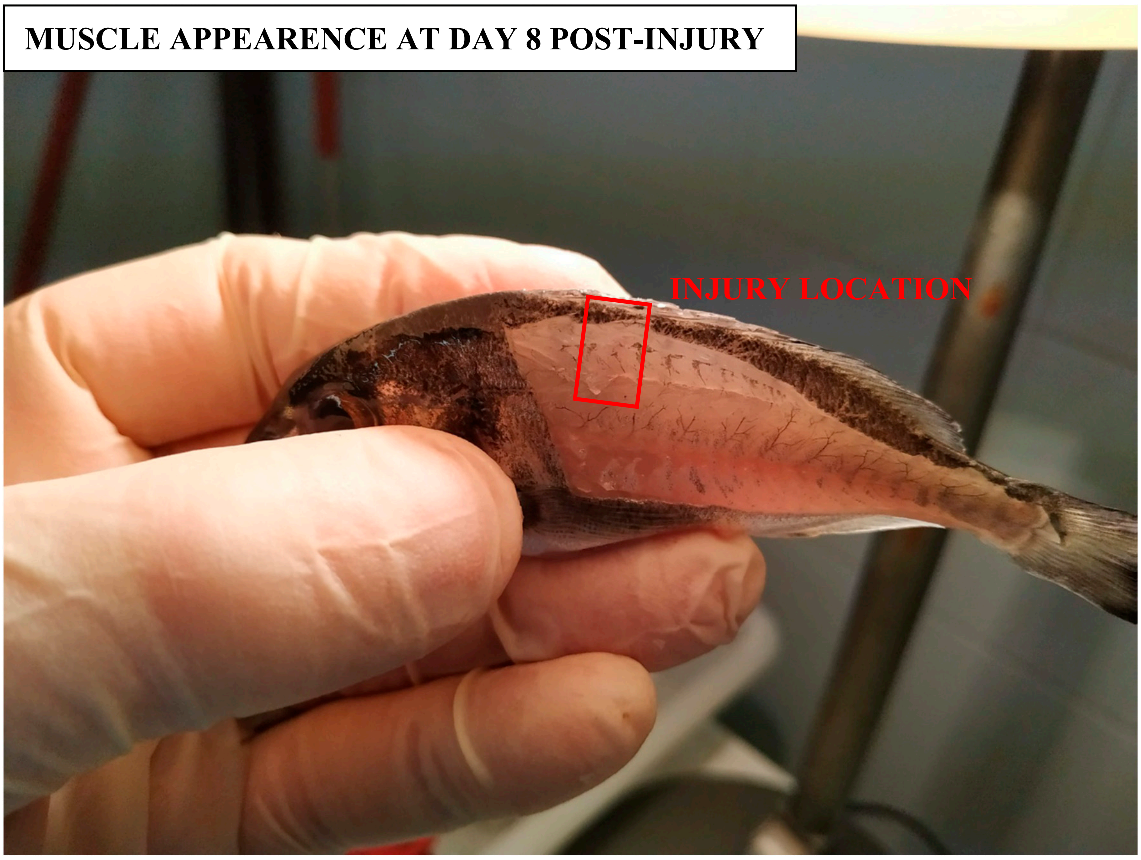
MUSCLE APPEARENCE AT DAY 8 POST-INJURY
INJURY LOCATION

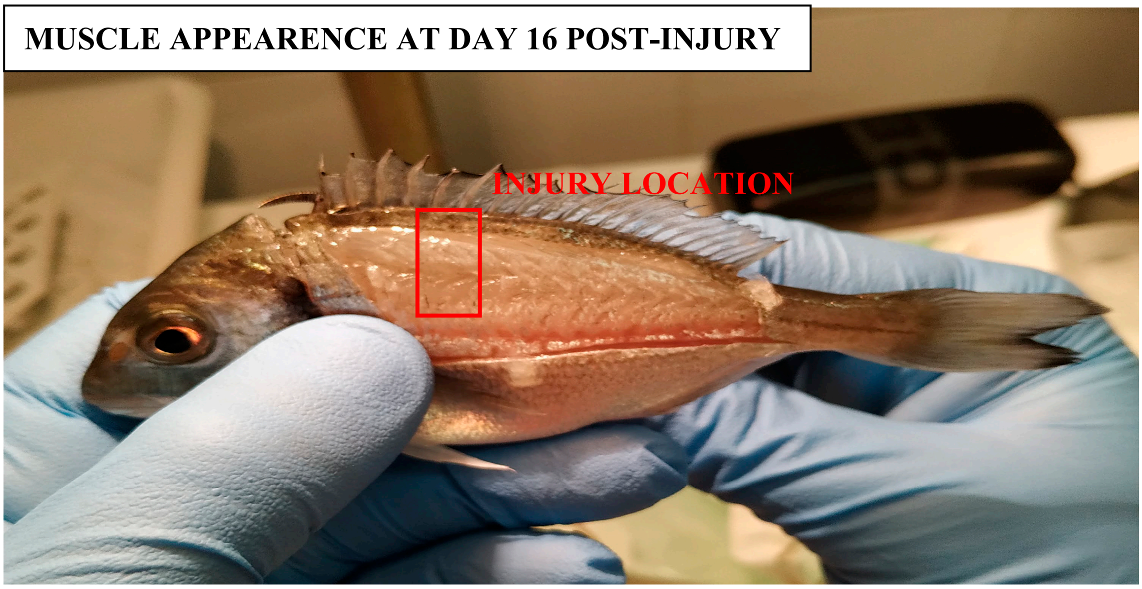
MUSCLE APPEARENCE AT DAY 16 POST-INJURY
INJURY LOCATION

WHITE MUSCLE SAMPLING PROCEDURE

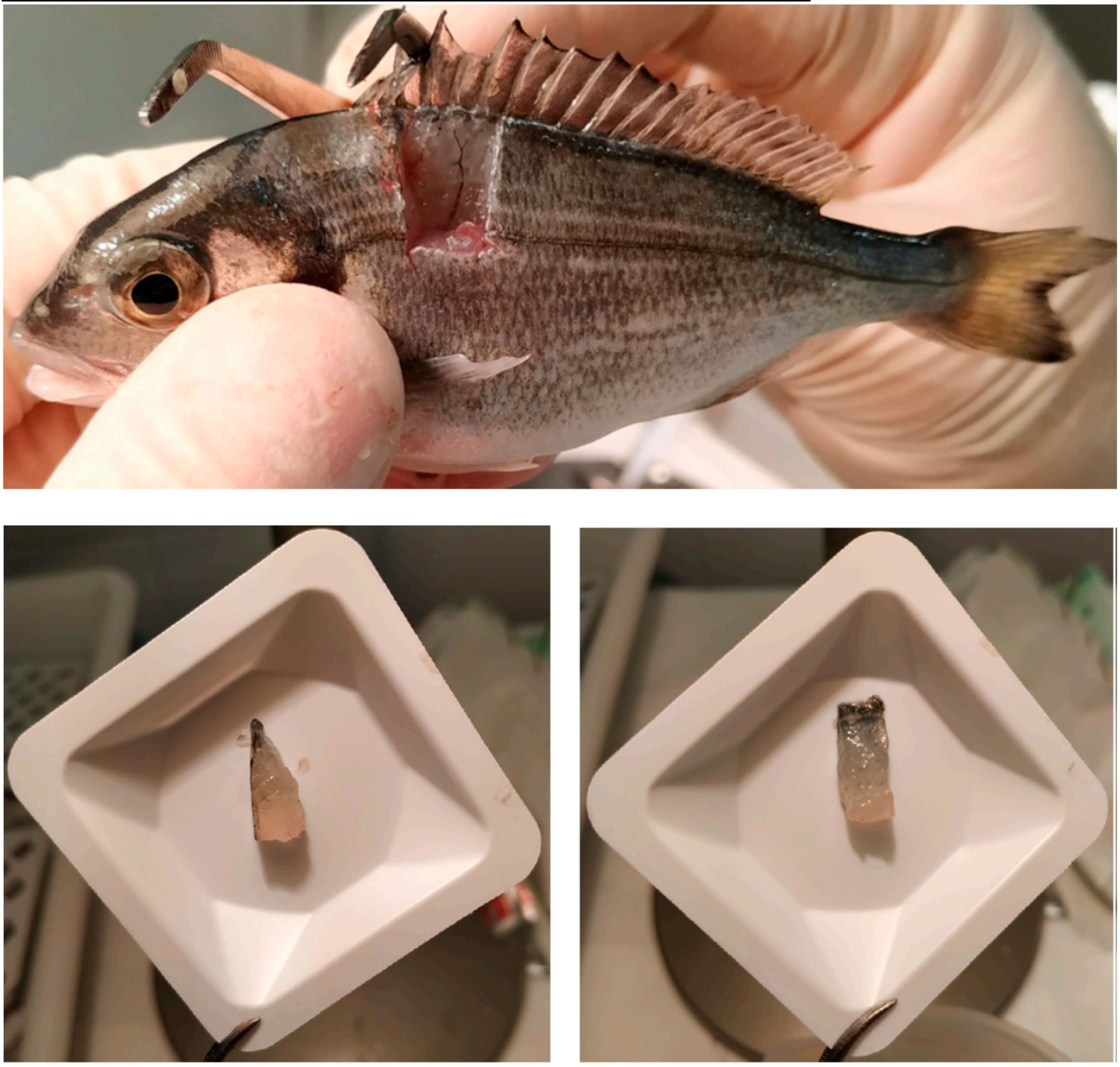

Supplement: Supplementary file 1 [file ijms-23-14639-s001.zip › ijms-1979213-supplementary.pdf]
